# Frequencies of *CYP2C19* Polymorphisms in Mexican Mestizo Patients with Coronary or Cerebral Atherosclerosis and Their Association with Residual Platelet Activity on Clopidogrel Treatment

**DOI:** 10.3390/biomedicines14091970

**Published:** 2026-09-01

**Authors:** Raúl Izaguirre-Ávila, Beatriz Villegas-Torres, Evelyn Cortina de La Rosa, Juan Carlos Fernández-López, Jesús Manuel Ayala-Guerrero, Elias Vinicio Merlín-González, Claudia Lerma

**Affiliations:** 1 Departamento de Hematología, Instituto Nacional de Cardiología Ignacio Chávez, Mexico City 04480, Mexico; evelyncortina@yahoo.com.mx (E.C.d.L.R.); jesusayalaguerrero@hotmail.com (J.M.A.-G.); merlin_glz@hotmail.com (E.V.M.-G.); 2 Laboratorio de Diagnóstico Genómico, Instituto Nacional de Medicina Genómica, Mexico City 14610, Mexico; bevillegas@inmegen.gob.mx (B.V.-T.); jfernandez@inmegen.gob.mx (J.C.F.-L.); 3 Departamento de Biología Molecular, Instituto Nacional de Cardiología Ignacio Chávez, Mexico City 04480, Mexico; dr.claudialerma@gmail.com

**Keywords:** *CYP2C19*, clopidogrel resistance, coronary artery disease, platelet aggregation

## Abstract

**Background/Objectives**: There is wide variability in the individual response to clopidogrel, which depends on *CYP2C19* polymorphisms as well as on intrinsic and extrinsic platelet factors. Clopidogrel resistance has been related to an adverse evolution in patients with coronary artery disease. The aim of this study is to analyze the prevalence of *CYP2C19* polymorphisms in Mexican mestizo patients and their relationship with clopidogrel platelet response by means of platelet aggregation. **Methods**: Patients who experienced coronary artery disease (CAD) (*N* = 160) or stroke (*N* = 11) and received treatment with clopidogrel were analyzed for *CYP2C19* alleles *1, *2, *3, and *17. In total, 81 patients were tested for clopidogrel resistance by ADP-induced aggregation in platelet-rich plasma. *CYP2C19* polymorphism was expressed as ultrarapid-, normal-, rapid-, normal/intermediate, intermediate, and poor metabolizers, and the clopidogrel response was assessed by platelet aggregation. **Results**: The allelic frequencies were 9.6%, 0%, and 11.7% for CYP2C19*2, *3, and *17, respectively. The frequencies of *CYP2C19* genotypes were as follows: CYP2C19*1/*1 (62.6%); CYP2C19*1/*17 (19.9%); CYP2C19*1/*2 (12.3%); CYP2C19*2/*17 (2.3%); CYP2C19*2/*2 (2.3%); and CYP2C19*17/*17 (0.6%). Of 81 patients with platelet aggregation, 55.6% were responsive, and 44.4% were resistant to clopidogrel. Clopidogrel resistance had a non-significant trend of association with unfavorable genotypes for metabolism (*CYP2C19* *1/*2, *2/*17, and *2/*2) vs. favorable genotypes (*CYP2C19* *1/*1, *1/*17, and *17/*17), 64.7% vs. 39.1%, respectively (*p* = 0.059). **Conclusions**: The prevalence of *CYP2C19* polymorphisms in Mexican mestizo patients is similar to that of the general population. Clopidogrel resistance is a complex in vitro phenomenon that is not explained by *CYP2C19* polymorphisms alone.

## 1. Introduction

Among cardiovascular disorders, myocardial infarction (MI) remains one of the leading causes of mortality and constitutes the most severe clinical manifestation of CAD, frequently associated with sudden cardiac death. Clinically, MI is classified according to electrocardiographic findings into ST-segment elevation myocardial infarction (STEMI) and non-ST-segment elevation myocardial infarction (NSTEMI). Current global data indicate that more than 3 million individuals experience STEMI annually, while over 4 million experience NSTEMI. Although MI has historically had higher incidence rates in industrialized regions, it has also become increasingly common in low- and middle-income countries [1]. Antiplatelet drugs are the cornerstone of management for CAD, both in the acute phase and for secondary prevention. Clopidogrel decreases platelet aggregation and irreversibly binds to platelet *P2Y12* receptors, thereby inhibiting ADP-dependent aggregation. This thienopyridine is a prodrug that requires two oxidative processes catalyzed by cytochrome *P450* isoenzymes (*CYP1A2*, *CYP2B6*, *CYP2C19*, and *CYP3A4*) [2,3]. In the first stage, an intermediate metabolite, 2-oxo-clopidogrel, is produced, which in turn yields four isomeric metabolites in the second metabolic process. Only clopi-H4 shows antiplatelet activity in vivo [4]. There is wide variability in individual responses to clopidogrel, and in some patients, increased residual platelet activity is observed on platelet function tests despite treatment with this drug. This phenomenon is known as clopidogrel resistance and, more recently, as high on-treatment platelet hyperreactivity, and various studies suggest that it is a risk factor for the recurrence of cardiovascular events, death, and stent thrombosis [5,6]. The causes of clopidogrel resistance are divided between factors dependent on platelet function, the drug’s bioavailability, cytochrome *P450*, and the P2Y12 receptor polymorphisms [7], as well as extraplatelet factors that affect platelet reactivity. According to cytochrome *P450* C19 polymorphisms, which determine clopidogrel conversion of active metabolites, patients are classified in the following metabolizer groups: extensive metabolizers (EMs: CYP2C19*1/*1), also called wild type; rapid metabolizers (RM: CYP2C19*1/*17); intermediate metabolizers (IMs: CYP2C19*1/*2); ultra-rapid metabolizers (URs: CYP2C19*17/*17); normal/intermediate metabolizers (N/IMs: CYP2C19*2/*17); and poor metabolizers (PMs: CYP2C19*2/*2).

Marked variability in the distribution of pharmacogenomic variants influencing clopidogrel responsiveness has been identified among US Hispanic/Latino populations, underscoring the importance of these genetic differences for the design and interpretation of current and future pharmacogenetic investigations involving clopidogrel therapy. There are insufficient data from Latin American countries. The aims of this study were to analyze the prevalence of *CYP2C19* polymorphisms and to assess the association between polymorphisms and responsiveness to clopidogrel in a population of Mexican patients with CAD and cerebrovascular disease (CVD).

## 2. Materials and Methods

### 2.1. Study Design and Participants

This prospective, cross-sectional, observational study included 171 patients: 160 with CAD and 11 with CVD. Patient data were captured from 3 to 12 months after acute coronary syndrome, transient cerebral ischemia, or cerebral infarction. A survey was conducted on each patient, and their clinical records were reviewed to compile demographic data, diagnoses, cardiovascular risk factors, and associated diseases. Venous blood samples were collected to obtain DNA and analyze polymorphisms related to clopidogrel response. A subset of 81 patients underwent platelet aggregation testing to assess clopidogrel response, with “responsive” defined as maximal platelet aggregation (MPA) <60% and “resistant” as MPA ≥ 60%. The remaining 90 patients contributed only genotyping data. Platelet-function testing was performed 3 to 12 months after the acute event while patients were receiving chronic maintenance therapy with clopidogrel 75 mg once daily; at the acute event, a standard loading dose of 300–600 mg was administered. This time window was chosen deliberately, since during the first weeks to months after an acute event, systemic inflammation and increased platelet turnover transiently enhance platelet reactivity; assessment during the subsequent stable phase therefore provides a more representative measure of the response to clopidogrel [8].

All participants were Mexican mestizos, defined as self-identified individuals born in Mexico whose parents and grandparents were also born in Mexico, representing an admixture of Native American and European (mainly Spanish) ancestry.

### 2.2. Polymorphism Analysis

The buffy coat was separated and frozen until the moment of DNA separation and polymorphism analysis. Genomic DNA was isolated using the QIAamp ^®^ DNA Blood Mini and Maxi Kit (QIAGEN Inc., Valencia, CA, USA), and the DNA was eluted with TE buffer (Teknova Inc., Hollister, CA, USA). DNA samples were quantified in the NanoDrop ND-1000 spectrophotometer (NanoDrop Technologies, Inc., Wilmington, DE, USA) and were diluted in molecular biology-grade water. The genotypes of alleles *1, *2, *3, and *17 of *CYP2C19* were determined by an allelic discrimination assay with TaqMan probes (Applied Biosystems, Foster City, CA, USA) and TaqMan GT Master Mix (Applied Biosystems, Foster City, CA, USA), in a 7900HT Fast Real-Time PCR Thermal Cycler System (Applied Biosystems, Foster City, CA, USA). To assess analytical validity, the genotype was confirmed by bidirectional sequencing using locus-specific primers; we prepared a new dilution and reanalyzed genotyping in 30% of the samples.

### 2.3. Platelet Aggregation Tests

Of the 171 individuals studied, 81 underwent platelet aggregation testing to assess the responsiveness or resistance to clopidogrel treatment. Venous blood samples were drawn into 4.5 mL Vacutainer tubes containing 3.2% citrate in a 9:1 ratio. Platelet aggregation was performed within the first 2 h using a Chrono-Log Model 560 Whole Blood/Optical Lumi-Aggregometer (Ca^2+^)I (Chrono-Log Corp., Havertown, PA, USA), according to the Clinical and Laboratory Standards Institute (CLSI) published in 2008 [9]. Platelet-rich plasma (PRP) was obtained by centrifugation at 140× *g* for 5 min; autologous platelet-poor plasma (PPP) was obtained by centrifugation at 1000× *g* for 15 min, and it was used to adjust to a platelet count of 250 × 10^9^ platelets/L. To calculate the percentage of maximal platelet aggregation (MPA), maximal aggregation, expressed in arbitrary units, was used in a mix of samples obtained from healthy Mexican mestizo volunteer donors who had not taken any medication. Aggregation was induced using Chrono-Par adenosine diphosphate (ADP) (Chrono-Log Corp., Havertown, PA, USA) at 10 µM, following the manufacturer’s instructions. Clopidogrel response was established according to platelet aggregation reference values induced by ADP 10 µM (80–120%) in a healthy population with non-antiaggregant treatment. Responsiveness was defined as MPA < 60.0%, and resistance as MPA ≥ 60.0%.

### 2.4. Statistical Analysis

Continuous variables were tested for normal distribution using the Kolmogorov–Smirnov test and, according to their distribution, were compared using the Student’s *t*-test, Mann–Whitney U test, or Kruskal–Wallis test. Differences in categorical proportions by sex and clopidogrel response were analyzed with the chi-squared test and, when necessary, Fisher’s exact test. A *p*-value < 0.05 was considered statistically significant. Allele frequencies were calculated using PLINK (version 1.07; Purcell S., Boston, MA, USA). All statistical analyses were performed using R (version 4.5.3; R Foundation for Statistical Computing, Vienna, Austria) within the RStudio integrated development environment (version 2026.04.0; Posit, PBC, Boston, MA, USA).

## 3. Results

### 3.1. Study Population Characteristics

The study cohort was predominantly male, with a male-to-female ratio close to 6:1, and most patients had coronary rather than cerebrovascular disease (Table 1). Median age was similar in men and women. Dyslipidemia was the most frequent cardiovascular risk factor and obesity the least frequent. A history of smoking was the only characteristic that differed significantly between sexes, being markedly more common in men; the distribution of coronary and cerebrovascular disease was similar in men and women.

Among the clinical presentations of coronary artery disease, ST-segment elevation myocardial infarction predominated, whereas unstable angina was the least frequent (Table 2). Percutaneous transluminal coronary angioplasty was the main revascularization strategy and was accompanied by stent placement in almost all treated patients, while coronary artery bypass grafting was performed far less often; stenting was significantly more frequent in men than in women. At the time of the study, most patients with coronary artery disease were receiving dual antiplatelet therapy with aspirin and clopidogrel, and the remainder were treated with aspirin alone.

### 3.2. Prevalence of CYP2C19 Genotypes

The wild-type normal-metabolizer genotype CYP2C19*1/*1 was by far the most common, followed by the rapid-metabolizer (*1/*17) and intermediate-metabolizer (*1/*2) genotypes (Table 3). The ultra-rapid-metabolizer genotype (*17/*17) was the least represented, and no patient carried a *3-containing genotype (*1/*3 or *3/*3), consistent with the near-absence of this allele outside East Asian populations. The genotype distribution did not differ significantly between men and women.

Table 4 shows that four *CYP2C19* alleles were characterized at the single-nucleotide level together with their known functional consequences for clopidogrel metabolism [10,11]. The functional *1 allele predominated. Among the variant alleles, the gain-of-function *17 (rs12248560) was the most frequent, slightly exceeding the loss-of-function *2 (rs4244285), whereas the loss-of-function *3 allele (rs4986893) was undetected. The relatively high frequency of the *17 allele is noteworthy, since it defines a rapid or ultra-rapid metabolizer profile associated with greater exposure to the active metabolite and, potentially, an increased bleeding risk [10,11].

### 3.3. Association Between Genotype and Clopidogrel Response

Table 5 compares the clinical and demographic profile of patients who underwent light-transmission aggregometry with those who did not. Patients in the aggregometry group were significantly older and had a higher prevalence of type 2 diabetes and hypertension, and all of them had confirmed coronary artery disease. The two groups were otherwise comparable, with no significant differences in smoking, dyslipidemia, or obesity.

Table 6 contrasts clopidogrel-responsive and clopidogrel-resistant patients. The two groups were comparable in age and in every cardiovascular risk factor examined. The proportion of non-responders to clopidogrel was higher among women, although this difference did not reach statistical significance and should be interpreted cautiously. None of the hemostatic or inflammatory biomarkers (factor VIII activity, fibrinogen, C-reactive protein, erythrocyte sedimentation rate, and leukocyte count) differed significantly between groups, indicating that they did not account for the resistant phenotype.

### 3.4. Differences in Maximal Platelet Aggregation According to Genotypes

**Figure 1:**
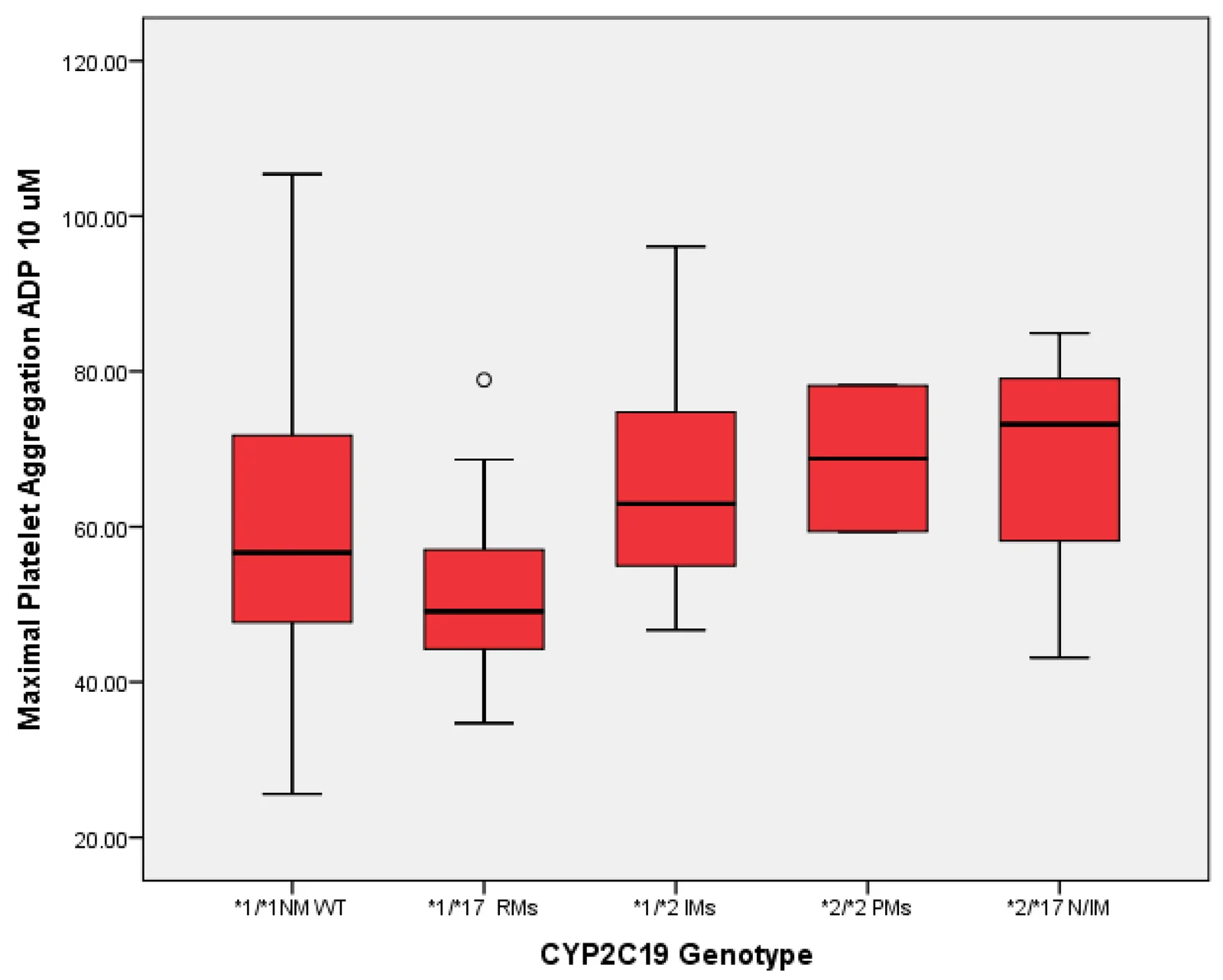
Distribution of maximal ADP-induced platelet aggregation across *CYP2C19* metabolizer genotypes (Kruskal–Wallis test, *p* = 0.098). Boxes represent the median and interquartile range, and whiskers represent the range; higher values indicate weaker clopidogrel-mediated platelet inhibition. The CYP2C19*17/*17 genotype is not shown; it was identified in only one patient, who had a maximal platelet aggregation of 38% and was classified as clopidogrel-responsive. Abbreviations: NM, normal metabolizer; RM, rapid metabolizer; IM, intermediate metabolizer; PM, poor metabolizer; N/IM, normal/intermediate metabolizer; MPA, maximal platelet aggregation.

Figure 1 displays the distribution of maximal ADP-induced platelet aggregation across the *CYP2C19* genotype groups. Residual aggregation was lowest in the single ultra-rapid metabolizer (*17/*17) and in rapid metabolizers (*1/*17) and tended to be higher in carriers of the *2 loss-of-function allele (intermediate, normal/intermediate, and poor metabolizers), consistent with a gradient of progressively reduced clopidogrel activation. Maximal platelet aggregation showed a numerical tendency to differ across *CYP2C19* genotype groups, but the overall comparison did not reach statistical significance (Kruskal–Wallis *p* = 0.098).

### 3.5. Analysis of CYP2C19 Genotype Groups

Table 7 details clopidogrel response for each *CYP2C19* genotype among the 81 patients who underwent platelet aggregometry. Resistance was concentrated in carriers of loss-of-function genotypes, being most pronounced in intermediate (*1/*2), normal/intermediate (*2/*17), and poor (*2/*2) metabolizers, whereas rapid metabolizers (*1/*17) were overwhelmingly responsive. Notably, a substantial proportion of normal metabolizers (*1/*1) were also resistant, showing that an adequate metabolic genotype does not guarantee an effective antiplatelet response. The overall differences across genotypes did not reach statistical significance. These genotype-specific patterns should be interpreted with caution, since the carriers of the least frequent genotypes were very few (ultra-rapid *17/*17, *n* = 1; poor *2/*2, *n* = 2; normal/intermediate *2/*17, *n* = 3); the corresponding estimates of residual platelet reactivity are therefore imprecise, the pairwise comparisons are underpowered, and the trend toward higher aggregation in loss-of-function carriers should be regarded as hypothesis-generating.

Table 8 groups patients by the functional impact of their *CYP2C19* variants, contrasting carriers of favorable alleles (favorable *CYP2C19* alleles: *1/*1, *1/*17, and *17/*17) with carriers of unfavorable loss-of-function alleles (unfavorable *CYP2C19* alleles: *1/*2, *2/*17, and *2/*2). Responsive patients were predominantly found in the favorable group, whereas resistance was numerically more frequent in the unfavorable group. Although this difference did not reach statistical significance, it points to a clinically meaningful association between loss-of-function genotypes and reduced clopidogrel response (odds ratio for resistance in unfavorable vs. favorable metabolizers, 2.86; 95% CI 0.94–8.71).

## 4. Discussion

In the present study, we found that the prevalence of *CYP2C19* cytochrome polymorphisms in our population is similar to that reported in other racial groups. For instance, a study conducted in a Vietnamese population found a clopidogrel resistance rate of 29.9% and an adverse event incidence of 15.9%. The frequencies of CYP2C19*2 and CYP2C19*3 were higher among patients with clopidogrel resistance than among those without [12].

In another study by Nawaz et al., conducted in 390 Pakistani patients receiving clopidogrel for ischemic heart disease, 50.24% were homozygous for the CYP2C19*1/*1 polymorphism, 40.8% were heterozygous for *1/*2, and 9% were homozygous for *2/*2. Platelet aggregation studies showed that 157 patients (80.1%) were clopidogrel responders and 39 (19.9%) were clopidogrel resistant among GG carriers (normal metabolizers); 118 (74.2%) were responders, and 41 (25.8%) were resistant among GA carriers (intermediate metabolizers); and 18 (51.4%) were responders, and 17 (48.6%) were resistant among AA carriers (poor metabolizers) (*p* = 0.001). The mean inter-group platelet aggregation was significantly different (*p* = 0.025). The allele frequency of the dominant allele *1 and the polymorphic variant allele *2 was 0.706 (70.6%) and 0.294 (29.4%), respectively [13].

In a distinct cohort by Lodhi et al., involving 668 Indian patients with acute coronary syndrome undergoing percutaneous coronary intervention, polymorphism testing was performed, and the results were compared with those of 143 patients who did not undergo platelet function testing to evaluate the association with clopidogrel metabolism. Clopidogrel resistance was observed in 54.6% of patients, and clinical outcomes were monitored. An adverse *CYP2C19* polymorphism was strongly associated with clopidogrel resistance and unfavorable clinical outcomes. These findings indicate that patients with an inadequate clopidogrel response have a significantly higher risk of stent thrombosis and acute myocardial infarction [14].

Similar results have been reported in patients with peripheral arterial disease. Zhang et al. studied 345 Chinese patients who underwent revascularization for peripheral arterial disease over a 5-year period to identify risk factors for recurrent ischemic events. Platelet reactivity and *CYP2C19* genotypes were determined. Among other risk factors, polymorphisms associated with intermediate and poor metabolism were identified as independent risk factors for new ischemic events. Decreased clopidogrel metabolism increased platelet reactivity, which was associated with a higher risk of ischemic events [15]. The frequency of the loss-of-function *2 allele in our cohort was comparable to that reported in other non-East Asian populations [16].

Similar patterns have been described in Italian [17] and Colombian [18] cohorts, in which the *1/*1 genotype predominates. In contrast, loss-of-function genotypes are considerably more frequent in East Asian populations, such as the Indian cohort [19], with intermediate frequencies described in Russian populations [20].

The pharmacogenetic characterization of *CYP2C19* polymorphisms in the Mexican mestizo population has been addressed progressively over the past decade through a series of complementary studies. However, evidence on *CYP2C19* pharmacogenomics and clopidogrel responsiveness remains limited.

Vargas-Alarcón et al. [21] described the allele frequencies of CYP2C19*2 and *3 (among other pharmacogenes) in 269 healthy Mexican mestizos, finding that the *3 allele was undetectable in this population and that the overall pharmacogene distribution differed significantly from other ethnic groups. Our findings are partially confirmatory of that work: we corroborate the near-absence of the *3 allele and the low frequency of the *2 loss-of-function allele in the Mexican mestizo population. However, our study extends theirs by analyzing patients with established atherosclerotic disease receiving clopidogrel rather than healthy individuals, by additionally characterizing the gain-of-function *17 allele, and by relating genotype to a functional measure of residual platelet reactivity.

The only published studies that have specifically evaluated *CYP2C19* genotype in the context of clopidogrel therapy are those of Viveros et al. [22], who assessed platelet reactivity by VASP phosphorylation in 90 PCI patients from Michoacán, and Cedillo-Salazar et al. [23], who confirmed the association between the CYP2C19*2 allele and reduced platelet inhibition in 102 high-cardiovascular-risk patients from northeastern Mexico using the VerifyNow P2Y12 assay. Both studies were restricted to the loss-of-function *2 variant and did not address the gain-of-function *17 allele. Taken together, these studies have established a consistent pattern of interindividual variability in clopidogrel response in the Mexican population, with a convergent frequency of the homozygous loss-of-function genotype (*2/*2) of 2–4% (Table 9). Beyond the frequencies of individual alleles, the admixed nature of the Mexican mestizo population—a blend that mainly reflects Native American and European ancestry—plays a crucial role in interpreting the varying frequencies of pharmacogenetic factors and may underlie differences in diagnosis, prognosis, and therapeutic response in clinical care, revealing a notable degree of genetic diversity [24]. *CYP2C19* single-nucleotide polymorphisms account for only part of the clinical response to clopidogrel, and contributions from additional genes, complex phenotypes, and environmental factors are still required to increase the statistical power of pharmacogenetic analyses in this predominantly mestizo population; this underscores the value of multidisciplinary research for improving clinical practice.

The present study extends the pharmacogenetic characterization of clopidogrel metabolism in the Mexican population by simultaneously genotyping four *CYP2C19* alleles—*1, *2, *3, and *17—in a cohort of 171 patients with coronary artery disease or cerebrovascular disease. This approach enabled phenotypic classification into six metabolizer categories, encompassing both loss- and gain-of-function variants, thereby offering a more comprehensive view of interindividual pharmacokinetic variability than single-allele approaches used in prior investigations in this population. Notably, rapid and ultrarapid metabolizers—carriers of the CYP2C19*17 gain-of-function allele—accounted for 20.5% of the cohort, a clinically meaningful subgroup given that enhanced metabolic activation of clopidogrel is associated with greater exposure to the active metabolite and, consequently, a potential increase in bleeding risk. From a pharmacodynamic standpoint, grouping patients according to their favorable or unfavorable genotypic profile for drug metabolism revealed higher clopidogrel resistance rates in carriers of an unfavorable metabolic profile, as assessed by light transmission aggregometry (39.1% vs. 64.7%; *p* = 0.059), a difference that approached but did not reach statistical significance. The analytical robustness of these findings is further supported by bidirectional sequencing confirmation in 30% of the samples, which reinforces the internal validity of the genotypic classification applied.

The most frequent polymorphism identified in our study was CYP2C19*1/*1, corresponding to the normal metabolizer phenotype, followed by CYP2C19*1/*17 (rapid metabolizer) and CYP2C19*1/*2 (intermediate metabolizer). The first two polymorphisms were present in 82.5% of our study population (Table 3). This indicates that most patients receiving clopidogrel in our setting carry a genetic substrate adequate to produce the drug’s active metabolite. The CYP2C19*2/*2 polymorphism, corresponding to the poor metabolizer phenotype, was identified in only 2.3% of patients, suggesting that a minority of the clopidogrel-treated population may have subtherapeutic concentrations of the active metabolite.

The prevalence of polymorphisms associated with poor clopidogrel metabolism (CYP2C19*1/*2, CYP2C19*2/*17, and CYP2C19*2/*2) was higher in the resistant group compared with the sensitive group (30.6% vs. 13.3%) (Table 8). Among the 36 individuals with clopidogrel resistance, 22 (61.1%) patients carried the CYP2C19*1/*1 (normal metabolizer) polymorphism (Table 7) and exhibited platelet aggregation > 60%. This indicates that adequate platelet inhibition was not achieved. As these findings demonstrate, the presence of these polymorphisms does not account for all cases of resistance, supporting the notion that other contributing mechanisms exist.

In the present study, we found no significant differences in the prevalence of diabetes, hypertension, smoking, dyslipidemia, obesity, factor VIII concentration, or fibrinogen levels between patients with clopidogrel resistance and those with sensitivity. There was also no difference in inflammatory markers: C-reactive protein, erythrocyte sedimentation rate, or white blood cell count. It is reasonable to assume that other factors contribute to clopidogrel resistance in our patients. Platelet hyperreactivity in cardiovascular disease has been well described [25,26,27]. Elevated platelet reactivity during clopidogrel therapy has also been reported as a predictor of cardiovascular death, recurrent myocardial infarction, and stent thrombosis [28]. In a study by Wu et al. involving 139 patients with ischemic cerebrovascular disease treated with clopidogrel, participants were classified into two groups based on adenosine diphosphate (ADP)-induced platelet aggregation. Clopidogrel resistance was defined as an inhibition rate <30%, while the remaining patients were classified as clopidogrel responders. Of the total, 58.27% were clopidogrel responders, and 41.7% exhibited resistance. Female carriers of CYP2C19*2/*3 demonstrated clopidogrel resistance as an independent factor. Clopidogrel resistance was associated with stroke progression [29]. Clopidogrel resistance has lower predictive value for coronary stent thrombosis [30,31]. Other studies have found that poor-metabolizer polymorphisms (CYP2C19*2/*2) have low predictive power for recurrent adverse events [32]. More recently, the potential role of platelet function testing in the acute management of stroke with antiplatelet agents, including Cangrelor (which has a short half-life), has been considered [33].

Furthermore, the concept of antiplatelet resistance has evolved, with definitions varying. From a clinical standpoint, it refers to the recurrence of cardiovascular events despite antiplatelet therapy; however, the laboratory-based definition has attracted greater attention. From the perspective of platelet function testing, resistance has been defined as elevated residual platelet reactivity observed in patients during clopidogrel treatment. This is a complex phenomenon involving numerous intrinsic and extrinsic platelet-related factors.

Polymorphisms associated with poor metabolism have a low prevalence and do not account for all cases of resistance. The study by Sun et al. concluded that the CYP2C19*2 gene polymorphism is associated with clopidogrel resistance, as measured by platelet function assay, and that the variant allele (A vs. G) increases clopidogrel resistance. This finding may be used to guide individualized antiplatelet therapy with clopidogrel. Given the limitations of that study and the multiple definitions of clopidogrel resistance, increasingly in-depth investigations into the *CYP2C19* *2 gene polymorphism and antiplatelet therapy are warranted [34].

Other antiplatelet drugs that produce more potent and more rapid platelet inhibition include prasugrel and ticagrelor. The TRITON trial demonstrated that prasugrel is particularly beneficial in patients with type 2 diabetes mellitus and other risk factors for recurrent cardiovascular events [35]. The ACAPULCO and JUMBO trials have shown that prasugrel produces greater platelet inhibition than clopidogrel [36,37].

The importance of identifying high-risk patients for recurrent events—such as individuals with type 2 diabetes mellitus, extensive coronary artery disease, multiple coronary stents, stent thrombosis, or poor clopidogrel metabolism—lies in the fact that these patients may be candidates for more potent antiplatelet agents. *CYP2C19* polymorphism testing could serve as an additional tool for identifying patients at high risk of adverse vascular events and for exploring alternative treatment options. Clopidogrel will continue to play a fundamental role in the management of ischemic heart disease, particularly in patients without other factors that may interfere with an adequate therapeutic response [38].

The 2022 Clinical Pharmacogenetics Implementation Consortium (CPIC) guideline for clopidogrel therapy recommends that, in patients with acute coronary syndrome undergoing percutaneous coronary intervention who carry a hypometabolizer *CYP2C19* polymorphism, clopidogrel should be avoided and an alternative antiplatelet therapy should be considered, provided there is no contraindication [10,39]. Current guidelines recommend individualizing antiplatelet therapy according to patient risk, and the concept of de-escalation of antiplatelet intensity has gained increasing relevance. The appropriate use and monitoring of antiplatelet agents require the application of risk stratification scales, clear definitions of hemorrhagic and ischemic events, and careful selection of the pharmacological agent. Given the substantial evidence from published studies and the availability of more potent antiplatelet agents, *CYP2C19* genotyping may prove useful in guiding antiplatelet therapy selection in selected cases.

A substantial proportion of clopidogrel-resistant patients carried the wild-type CYP2C19*1/*1 genotype (46.8% of *1/*1 carriers were resistant), which indicates that *CYP2C19* polymorphisms alone cannot fully explain residual platelet reactivity in this population. Several additional factors may contribute to clopidogrel resistance, including other genetic variants involved in drug absorption, transport, and bioactivation (*ABCB1*, *PON1*, *CES1*, and *P2RY12*); drug–drug interactions, particularly with proton-pump inhibitors and other *CYP2C19* inhibitors; diabetes-related platelet hyperreactivity; systemic inflammation; and medication non-adherence. These mechanisms, together with the loss- and gain-of-function *CYP2C19* alleles, are likely to act jointly in determining the individual antiplatelet response. In this sense, the genotype-level associations reported here are best regarded as hypothesis-generating: they identify plausible directions for future research rather than definitive effects and require confirmation in larger, adequately powered cohorts before they can guide clinical decisions [39,40].

The present study has several limitations. First, it was conducted at a single tertiary referral center and included a relatively small and predominantly male cohort, which limits the statistical power of the analyses and the extrapolation of the findings to the general Mexican mestizo population. Second, platelet function testing was performed in only 81 of the 171 genotyped patients; this subgroup was older and had a higher prevalence of type 2 diabetes and hypertension, and all of its members had confirmed coronary artery disease, so the aggregometry sample was not randomly selected and may be affected by selection bias. Third, the number of carriers of the less frequent genotypes was very small, particularly ultra-rapid (CYP2C19*17/*17), poor (CYP2C19*2/*2), and normal/intermediate (CYP2C19*2/*17) metabolizers; consequently, the corresponding estimates of residual platelet reactivity are imprecise, the pairwise comparisons are underpowered, and the difference in the rate of resistance between favorable and unfavorable metabolizer groups did not reach conventional statistical significance. Fourth, genotyping was restricted to the *2, *3, and *17 alleles, and other loss-of-function variants, as well as additional genes involved in the absorption, transport, and bioactivation of clopidogrel, were not analyzed, which may have resulted in misclassification of the predicted metabolizer phenotype. Fifth, platelet reactivity was assessed by light transmission aggregometry at a single time point, with a single agonist concentration and a single cut-off value; since no universally accepted definition of clopidogrel resistance exists, the observed proportion of resistant patients may vary according to the assay and the threshold applied. Moreover, adherence to treatment was not confirmed by direct quantification of the active metabolite, and other factors known to modify the response to clopidogrel, such as drug–drug interactions, inflammatory status, and metabolic comorbidity, could not be fully controlled. Finally, the cross-sectional design and the lack of clinical follow-up preclude any inference regarding the association between *CYP2C19* genotype, residual platelet reactivity, and the occurrence of ischemic or hemorrhagic events; prospective studies with clinical endpoints are required to establish the prognostic usefulness of pharmacogenetic testing in this population.

## 5. Conclusions

Our data demonstrate that most of the study population carries *CYP2C19* polymorphisms that would enable an adequate response to clopidogrel. The combination of pharmacogenetic testing and platelet function assays could identify high-risk patient subgroups in whom more potent antiplatelet agents may be indicated. To date, no laboratory-derived level of platelet inhibition has been established to reliably predict favorable clinical outcomes or identify patients at the highest risk of recurrence. Platelet function testing and genotyping may serve as complementary tools to support clinical decision-making.

## Figures and Tables

**Table 1:** Baseline clinical and demographic characteristics of the 171 patients with atherosclerotic cardiovascular disease receiving clopidogrel.

Variable	Total (*N* = 171)	Men (*N* = 147)	Women (*N* = 24)	*p*-Value
Age (years)	56.0 (50–61.0)	56.0 (50–61)	59.0 (48–67)	0.560
Former or current smoker	98 (57.3%)	94 (63.9%)	4 (16.7%)	<0.001
Type 2 diabetes	47 (27.5%)	41 (27.9%)	6 (25.0%)	1.000
Hypertension	106 (62.0%)	87 (59.2%)	19 (79.2%)	0.062
Dyslipidemia	112 (65.5%)	94 (63.9%)	18 (75.0%)	0.359
Obesity	45 (26.3%)	41 (27.9%)	4 (16.7%)	0.322
CVD	11 (6.4%)	9 (6.1%)	2 (8.3%)	0.654
CAD	160 (93.6%)	138 (93.9%)	22 (91.7%)	0.654

Continuous variables are shown as median (25th–75th percentile) and categorical variables as *N* (%); CAD, coronary artery disease; CVD, cerebrovascular disease.

**Table 2:** Clinical presentation and revascularization treatment of the 160 patients with coronary artery disease.

CAD	Total (*N* = 160)	Men (*N* = 138)	Women (*N* = 22)	*p*-Value
CAD type				0.093
STEMI	106 (66.2%)	96 (69.6%)	10 (45.4%)	
Non-STEMI	22 (13.7%)	18 (13.0%)	4 (18.2%)	
Unstable Angina	13 (8.1%)	10 (7.2%)	3 (13.6%)	
Stable Angina	19 (11.9%)	14 (10.1%)	5 (22.7%)	
CABG	27 (16.9%)	23 (16.7%)	4 (18.2%)	0.860
Stent	117 (73.1%)	107 (77.5%)	10 (45.4%)	0.002
Balloon	13 (8.2%)	12 (8.7%)	1 (4.5%)	0.493

Values are *N* (%); the CAD-type *p*-value refers to the overall distribution of the four presentations between sexes; CABG, coronary artery bypass grafting; STEMI, ST-segment elevation myocardial infarction; Non-STEMI, non-ST-segment elevation myocardial infarction.

**Table 3:** Distribution of *CYP2C19* metabolizer genotypes across the 171-patient cohort, stratified by sex (*p* = 0.850 between sexes).

Polymorphisms	Total (*N* = 171)	Men (*N* = 147)	Women (*N* = 24)
Normal Metabolizer (Wild Type) (EM) CYP2C19*1/*1	107 (62.6%)	90 (61.2%)	17 (70.8%)
Rapid Metabolizer (RM) CYP2C19*1/*17	34 (19.9%)	30 (20.4%)	4 (16.7%)
Intermediate Metabolizer (IM) CYP2C19*1/*2	21 (12.3%)	19 (12.9%)	2 (8.3%)
Ultra-Rapid Metabolizer (UR) CYP2C19*17/*17	1 (0.6%)	1 (0.7%)	0 (0%)
Normal/Intermediate Metabolizer (N/IM) CYP2C19*2/*17	4 (2.3%)	3 (2.0%)	1 (4.2%)
Poor Metabolizer (PM) CYP2C19*2/*2	4 (2.3%)	4 (2.7%)	0 (0%)

Values are *N* (%). Genotype-defined metabolizer status determines the capacity to bioactivate clopidogrel. * The asterisk (*) denotes a “star allele,” the standardized haplotype nomenclature used to designate *CYP2C19* gene variants (e.g., *1, *2, *3, *17); each star allele represents a specific haplotype with a defined effect on enzyme function.

**Table 4:** *CYP2C19* allele frequencies and their functional consequences for clopidogrel bioactivation.

*CYP2C19* Allele	Allelic Frequency *N* (%)	SNP Reference	Enzyme Effect on Clopidogrel Metabolism
*1	269 (78.7%)	N/A	Normal
*2	33 (9.6%)	rs4244285	Inactive
*3	0 (0%)	rs4986893	Inactive
*17	40 (11.7%)	rs12248560	Increase

The *1 allele is the reference (wild-type) allele, associated with normal, fully functional enzyme activity; it is assigned by default when none of the variant alleles tested are detected, the *2 and *3 alleles abolish enzyme activity, and the *17 allele increases it, respectively decreasing and increasing the formation of the active metabolite [10,11]. Values are *N* (%). SNP, single-nucleotide polymorphism; N/A, not applicable.

**Table 5:** Comparison of clinical and demographic characteristics between patients who did and did not undergo platelet aggregometry.

Variable	Aggregometry (*N* = 81)	No Aggregometry (*N* = 90)	*p*-Value
Age (years)	57.0 (52–65.0)	53.5 (45.0–59.0)	0.013
Former or current smoker	48 (59.2%)	50 (55.5%)	0.625
Type 2 diabetes	29 (35.8%)	18 (20.0%)	0.021
Hypertension	57 (70.4%)	49 (54.4%)	0.032
Dyslipidemia	50 (61.7%)	62 (68.8%)	0.325
Obesity	23 (28.4%)	22 (24.4%)	0.558
CAD	81 (100.0%)	79 (87.7%)	0.001

Continuous variables are shown as medians (25th–75th percentile) and categorical variables as *N* (%).

**Table 6:** Clinical, demographic, and laboratory characteristics of clopidogrel-responsive versus clopidogrel-resistant patients (*N* = 81).

Variable	Responsive (*N* = 45)	Resistant (*N* = 36)	*p*-Value
Age (years)	57.3 ± 8.37	57.9 ± 11.2	0.093
Sex			0.083
Men	44 (97.8%)	31 (86.1%)	
Women	1 (2.2%)	5 (13.9%)	
Former or current smoker	26 (57.8%)	22 (61.1%)	0.762
Type 2 diabetes	13 (28.9%)	16 (44.4%)	0.147
Hypertension	34 (75.6%)	23 (63.9%)	0.253
Dyslipidemia	26 (57.8%)	24 (66.7%)	0.413
Obesity	15 (33.3%)	8 (22.2%)	0.270
Factor VIII:c (%)	133 (43.40)	128 (40.17)	0.578
Fibrinogen (g/L)	3 (1.02)	3 (0.70)	0.229
C-reactive protein (mg/L)	3 (4.48)	3 (5.30)	0.534
Erythrocyte sedimentation rate (mm/h)	13 (11.45)	14 (11.31)	0.794
Leukocytes (×10^3^/µL)	6 (1.61)	6 (1.42)	0.387

Continuous variables are shown as mean ± SD or median (interquartile range) and categorical variables as *N* (%). Resistance was defined as maximal platelet aggregation ≥60%. Chi-squared test and Fisher’s exact test.

**Table 7:** Clopidogrel response by *CYP2C19* genotype among the 81 patients assessed by platelet aggregometry (*p* = 0.145 across genotypes, chi-squared test).

Polymorphisms	Total (*N* = 81)	Responsive (*N* = 45)	Resistant (*N* = 36)
Normal Metabolizer (Wild Type) (EM) CYP2C19*1/*1	47 (58.0%)	25 (55.5%)	22 (61.1%)
Rapid Metabolizer (RM) CYP2C19*1/*17	16 (19.8%)	13 (28.9%)	3 (8.3%)
Intermediate Metabolizer (IM) CYP2C19*1/*2	12 (14.8%)	4 (8.9%)	8 (22.2%)
Ultra-Rapid Metabolizer (UR) CYP2C19*17/*17	1 (1.2%)	1 (2.2%)	0
Normal/Intermediate Metabolizer (N/IM) CYP2C19*2/*17	3 (3.7%)	1 (2.2%)	2 (5.5%)
Poor Metabolizer (PM) CYP2C19*2/*2	2 (2.5%)	1 (2.2%)	1 (2.8%)

Values are *N* (%). Responsive, maximal platelet aggregation <60%; resistant, ≥60%. The asterisk (*) denotes a “star allele,” the standardized haplotype nomenclature used to designate *CYP2C19* gene variants (e.g., *1, *2, *3, *17); each star allele represents a specific haplotype with a defined effect on enzyme function.

**Table 8:** Clopidogrel response according to a favorable or unfavorable *CYP2C19* metabolic profile (*p* = 0.059; odds ratio for resistance 2.86, 95% CI 0.94–8.71).

Group	Phenotypes	Responsive (*N* = 45)	Resistant (*N* = 36)
Favorable *CYP2C19* alleles (*N* = 64)	*1/*1, *1/*17 and *17/*17	39 (86.7%)	25 (69.4%)
Unfavorable *CYP2C19* alleles (*N* = 17)	*1/*2, *2/*17 and *2/*2	6 (13.3%)	11 (30.6%)

Values are *N* (%). Favorable genotypes (*1/*1, *1/*17, *17/*17) preserve or enhance active metabolite formation, whereas unfavorable genotypes (*1/*2, *2/*17, *2/*2) carry a loss-of-function *2 allele.

**Table 9:** Comparative characteristics of indexed studies addressing *CYP2C19* polymorphisms and clopidogrel response in Mexican mestizo populations.

Reference	Target Population	*CYP2C19* Polymorphisms Studied	Clopidogrel Response Assessment
**Vargas-Alarcón G et al.** [21]	Healthy unrelated Mexican mestizo individuals. *N* = 269 INCICh, Mexico City	CYP2C19*2 (G681A) and *3 (G636A). Also: ABCB1 T3435C, CYP3A5 A6986G, P2RY12 G52T and C34T. CYP2C19*3 not detected in this population.	Not evaluated. Participants were healthy volunteers not receiving clopidogrel therapy.
**Viveros ME et al.** [22]	Patients undergoing PCI. *N* = 90 Michoacán, Mexico.	CYP2C19*2 (restriction enzyme SmaI). *2 carriers: 17%; AA homozygous: 3.9%.	VASP phosphorylation index (PRI) by flow cytometry. Non-responders: PRI ≥ 50%. Overall: 40% good responders; 38% with PRI 50–70%; 22% with PRI > 70%. AA genotype associated with higher platelet reactivity index.
**Cedillo-Salazar FR et al.** [23]	Patients with high cardiovascular risk receiving clopidogrel (75 mg/day). *N* = 102 Nuevo León, Mexico.	CYP2C19*2 (real-time PCR). G/G: 74.5%; G/A: 21.6%; A/A: 3.9%.	VerifyNow P2Y12 assay. Reduced effect: PRU ≥ 235. Allele carriers (G/A or A/A): PRU ≥ 235. CYP2C19*2 significantly associated with clopidogrel resistance (*p* = 0.003).
**Izaguirre-Ávila R et al. (Present study)**	Mexican mestizo patients with coronary or cerebral atherosclerosis *N* = 171 Mexico City.	CYP2C19*1, *2, *3, and *17 (TaqMan allelic discrimination; bidirectional sequencing validation in 30% of samples). Allele frequencies: *2 = 9.6%; *3 = 0%; *17 = 11.7%. Genotypes: *1/*1 (62.6%), *1/*17 (19.9%), *1/*2 (12.3%), *2/*17 (2.3%), *2/*2 (2.3%), *17/*17 (0.6%).	ADP-induced light-transmission aggregometry (*n* = 81); resistance defined as MPA ≥ 60%. Overall resistance 44.4%; higher in carriers of an unfavorable than a favorable *CYP2C19* profile (64.7% vs. 39.1%, *p* = 0.059).

* The asterisk (*) denotes a “star allele,” the standardized haplotype nomenclature used to designate *CYP2C19* gene variants (e.g., *1, *2, *3, *17); each star allele represents a specific haplotype with a defined effect on enzyme function.

## Data Availability

The datasets presented in this article are not readily available because they are subject to the Mexican law (NOM-004-SSA3-2012) on the protection of personal data contained in the clinical records of the patients studied. The data presented in this study are available from the corresponding author upon request.

## Abbreviations

The following abbreviations are used in this manuscript:

ADP	adenosine diphosphate
CABG	coronary artery bypass grafting
CAD	coronary artery disease.
CPIC	Clinical Pharmacogenetics Implementation Consortium.
CRP	C-reactive protein
CVD	cerebrovascular disease.
CYP	cytochrome P450
DNA	deoxyribonucleic acid
EM	normal (extensive) metabolizer
ESR	erythrocyte sedimentation rate
IM	intermediate metabolizer
IQR	interquartile range
MI	myocardial infarction.
MPA	maximal platelet aggregation
N/IM	normal/intermediate metabolizer
NSTEMI	non-ST-segment elevation myocardial infarction
PCI	percutaneous coronary intervention
PCR	polymerase chain reaction
PM	poor metabolizer
PPP	platelet-poor plasma
PRI	platelet reactivity index
PRP	platelet-rich plasma
PRUs	P2Y12 reaction units
PTCA	percutaneous transluminal coronary angioplasty
RM	rapid metabolizer.
SA	stable angina
SD	standard deviation
SNP	single-nucleotide polymorphism
STEMI	ST-segment elevation myocardial infarction
UA	unstable angina
UR	ultra-rapid metabolizer
VASP	vasodilator-stimulated phosphoprotein
